# Genome-Wide Characterization of *SNAC* Gene Family in Ten Cotton Species and Function Analysis of *GhSNAC3D* Under Cold Stress

**DOI:** 10.3390/plants14182894

**Published:** 2025-09-18

**Authors:** Jiliang Fan, Lu Meng, Faren Zhu, Jiahuan Niu, Ganggang Zhang, Junwei Wang, Zhonghui Li, Fei Wang, Hongbin Li

**Affiliations:** Key Laboratory of Oasis Town and Mountain-Basin System Ecology of Xinjiang Production and Construction Corps, Key Laboratory of Xinjiang Phytomedicine Resource and Utilization of Ministry of Education, College of Life Sciences, Shihezi University, Shihezi 832003, China; fanplanbeson@163.com (J.F.); mlulu@stu.shzu.edu.cn (L.M.); zhufaren163@163.com (F.Z.); 17599931126@163.com (J.N.); 19915233386@163.com (G.Z.); 18287018531@163.com (J.W.);

**Keywords:** cotton, *SNAC* gene family, VIGS, cold stress

## Abstract

The *SNAC* (Stress-responsive NAC) subfamily, a key branch of the conserved NAC transcription factor family, plays a central role in regulating plant stress response. However, systematic characterization of the *SNAC* family in cotton (*Gossypium* spp.) remains unclear. Employing a genome-wide screening approach, this study characterized 75 distinct *SNAC* transcription factor genes across ten *Gossypium* species, with tetraploid cottons harboring twice as many as their diploid progenitors. Phylogenetic analysis categorized the genes into three subgroups, with members of the same subgroup exhibiting conserved motif compositions and gene structures. Chromosomal localization revealed a conserved distribution pattern of *SNAC* genes between the Dt and At subgenomes in tetraploid cotton. Genomic collinearity analysis suggested that the primary driver of *SNAC* family expansion was segmental duplication. Promoter analysis predicted 2974 *cis*-regulatory elements, including cold- and hormone-responsive motifs, indicating their potential involvement in stress regulation. These *GhSNAC* genes indicated significant induced expressions under stress conditions, and *GhSNAC3D* exhibited the most significant up-regulated expression under low temperature stress. Genetic function studies displayed that VIGS-mediated *GhSNAC3D*-silencing significantly reduced the cold tolerance in cotton. This study systematically analyzed the genomic characteristics of the cotton *SNAC* family and functionally validated the molecular mechanism of *GhSNAC3D*-mediated cryogenic response, which lays a foundation for subsequent research on cold resistance in cotton and stress-resistant breeding.

## 1. Introduction

Cotton (*Gossypium*) is a globally cultivated economic crop of great importance [1]. Cultivated cotton is now widely distributed in high-altitude areas such as the northwest of Asia [2]. In these regions, cotton exhibits a heightened sensitivity to cooler temperatures, which leads to a lower germination rate, delayed sprouting, and weakened seedling vitality. This adversely impacts the growth and development of the cotton plants, ultimately causing a marked decline in both quality and yield [3,4]. Therefore, improving the cold resistance of cotton is an increasingly important goal.

Low temperatures represent an environmental challenge that significantly hinders the growth, productivity, and survival of plants [5,6]. As global climate change exacerbating temperature fluctuations, elucidating the detailed molecular mechanisms that govern the response to cold stress becomes increasingly important [7]. This understanding not only enhances our comprehension of fundamental biological processes but also opens avenues for improving the cold resistance of cash crops. Cold stress presents a considerable obstacle to cotton cultivation, especially in temperate regions and during the initial planting phases [8,9]. Cold stress, by causing physiological and molecular damage, leads to a reduction in germination rate and impaired seedling development, seriously threatening these initial growth stages [10]. After being subjected to cold stress, notable alterations occur in various physiological and biochemical indicators and the morphology of plant organelles [11]. Under low-temperature stress, numerous physiological and biochemical processes in plant cells undergo changes—for instance, the leakage of intracellular ions and structural alterations to various cellular components [12,13,14].

The *NAC* (*NAM*, *ATAF1/2*, *CUC2*) transcription factor family comprises a group of transcription factors that are specific to plants and play a crucial role in the responses to stress tolerance. The naming of the *NAC* transcription factor family originated from the *NAM (No Apical Meristem)* gene of *Pharbitis*, *ATAF1/2 (Arabidopsis Transcription Activator Factor 1/2)*, and *CUC2 (Cup-Shaped Cotyledon 2)* genes of *Arabidopsis thaliana* at first [15]. The *NAC* family’s members possess a highly conserved DNA-binding domain located at their N-terminus, consisting of a 150–160 amino acid residue region known as the NAM domain [16]. This domain exhibits high conservation during the evolutionary process. Studies have demonstrated that the *NAC* transcription factor family, unique to plants, is crucial for regulating growth and development processes, hormone responses, and resistance to stress [17,18,19,20]. For instance, the enhanced drought tolerance in rice is attributed to the overexpression of *OsNAC10* [21]; similarly, *SNAC1* and *ONAC045* overexpression contributes to improves drought and salt resistance in rice [22]. Additionally, the overexpression of *SNAC2*, *OsNAC5*, and *OsNAC6* enhances rice’s ability to resist cold, salt, drought, and hot stresses [23]. In *A. thaliana*, the overexpression of soybean *GmSNAC49* upregulates genes related to drought and ABA (Abscisic Acid) signaling pathways, thus increasing drought stress tolerance [24]. Furthermore, the overexpression of grapevine *VvNAC17* improves drought resistance in *A. thaliana* by upregulating genes associated with ABA and stress responses. The *Miscanthus MLNAC10* exerts the function of antioxidant enzymes by regulating the ABA signaling pathway, alleviates ROS-induced damage, and serves as an important regulatory factor in the tolerance to salt and drought stress [25].

The *SNAC* (*Stress-Responsive NAC*) subfamily, a significant component of the *NAC* family, plays a crucial role in the stress responses of plants. At present, comprehensive genome-wide analyses of the *SNAC* have been completed in several plants, including *A. thaliana*, *Populus trichocarpa*, *Casuarina equisetifolia*, *Musa acuminata*, and *Solanum lycopersicum* [26,27,28]. However, systematic recognition of the *SNAC* transcription factor family in cotton and research on its cold resistance function remain insufficient. This study achieved identification of the *SNAC* family in cotton through a genome-wide approach. Utilizing comprehensive genomic data, the *SNAC* gene family members in cotton were successfully identified [29]; then, through phylogenetic analysis, the chromosomal localization, gene structure, and collinear relationships of cotton *SNAC* genes were clarified. Additionally, investigations were conducted on their subcellular localization characteristics, expression patterns across different tissues and under various stress treatments, and phenotypic changes under cold stress. These research outcomes will enhance our comprehension of the mechanism by which *SNAC* family members function in cotton cold stress resistance, reveal the characteristics and diversity of cotton *SNAC* genes, and provide dual support for elucidating the mechanism of cotton cold stress response with evolutionary and functional molecular evidence.

## 2. Results

### 2.1. Characterization and Physicochemical Properties of SNAC Genes in Cotton

Through BLAST (v2.210) analysis, 5, 5, 10, 10, 10, 11, 10, 4, 5, and 5 *SNAC* genes were derived from the genomes of *G. herbaceum* (Ghe), *G. turneri* (Gtu), *G. barbadense* (Gb), *G. hirsutum* (Gh), *G. darwinii* (Gd), *G. mustelinum* (Gm), *G. tomentosum* (Gt), *G. thurberi* (Gth), *G. raimondii* (Gr), and *G. arboreum* (Ga), respectively (see Table S1). The NAM domain was confirmed in all candidate genes using the InterProScan and SMART tools. Notably, the number of *SNAC* genes in the tetraploid species *G. hirsutum* (Gh) is approximately twice that in its diploid ancestors, *G. raimondii* (Gr) and *G. arboreum* (Ga), indicating that most *SNAC* genes were retained during the polyploidization of Ga and Gr. Table S1 provides detailed physicochemical parameters of all SNAC proteins across the 10 *Gossypium* species, including gene ID, renamed ID, protein length, and molecular weight. The length of amino acid sequence for SNAC proteins ranges from 157 residues in *GthSNAC3* to 348 residues in *GdSNAC1A*. Their molecular weight ranges from 18.04 kDa for *GthSNAC3* to 38.69 kDa for *GdSNAC1A*, averaging at 34.22 kDa. Additionally, the isoelectric point (pI) ranges from 5.52 in *GtuSNAC4* to 9.72 in *GhSNAC3D*. The instability indices for all SNAC proteins range from 24 to 51, with an average grand average of hydropathicity (GRAVY) of less than 0. This suggests that these proteins are unstable, soluble, and hydrophilic (Table S1). These results reveal variations in molecular weight, the number of amino acids, and isoelectric point among *SNAC* family members, which may contribute to subtle functional variations.

### 2.2. Chromosomal Mapping of the SNAC Gene Family

**Figure 1:**
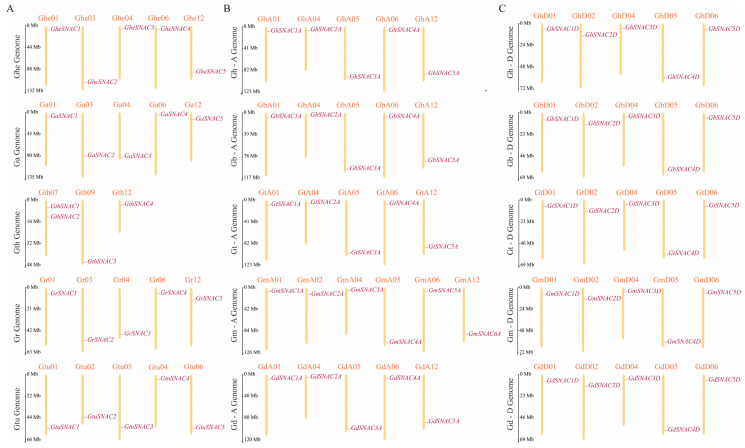
Localization of the *SNAC* on cotton chromosomes. (**A**) Localization of the *SNAC* within diploid cotton species (*G. herbaceum* (Ghe), *G. arboretum* (Ga), *G. thurberi* (Gth), *G. raimondii* (Gr), *G. turneri* (Gtu)); (**B**) Localization of the *SNAC* within the At subgenome of tetraploid cotton species (*G. tomentosum* (Gt), *G. barbadense* (Gb), *G. hirsutum* (Gh), *G. darwinii* (Gd), *G. mustelinum* (Gm)); (**C**) Localization of the *SNAC* within the Dt subgenome of tetraploid cotton species. Chromosomes are shaded in yellow, individual *SNAC* genes are marked in purple, and chromosome names are labeled in orange.

In this study, *SNAC* family members were designated sequentially according to their chromosomal positions (Table S1). Analysis revealed that most *SNAC* genes are distributed across 5 chromosomes, predominantly on chromosomes 1, 4, 5, 6, and 12. Notably, *SNAC* genes in the Gth genome are restricted to 3 chromosomes, with *GthSNAC1* and *GthSNAC2* co-localized on chromosome Gth07 (Figure 1). In the Gm-A subgenome, *SNAC* genes are distributed across 6 chromosomes, which may result from gene duplication and deletion events during evolution. Interestingly, as shown in Figure 1B,C, in the Dt and At subgenomes of tetraploid cotton, *SNAC* genes are basically located at the same position on different chromosomes. This observation further supports the high evolutionary conservation of the *SNAC* gene family.

### 2.3. Evolutionary Analysis of SNACs Coupled with Investigation of Conserved Motifs, Conserved Domains, and Gene Structures

**Figure 2:**
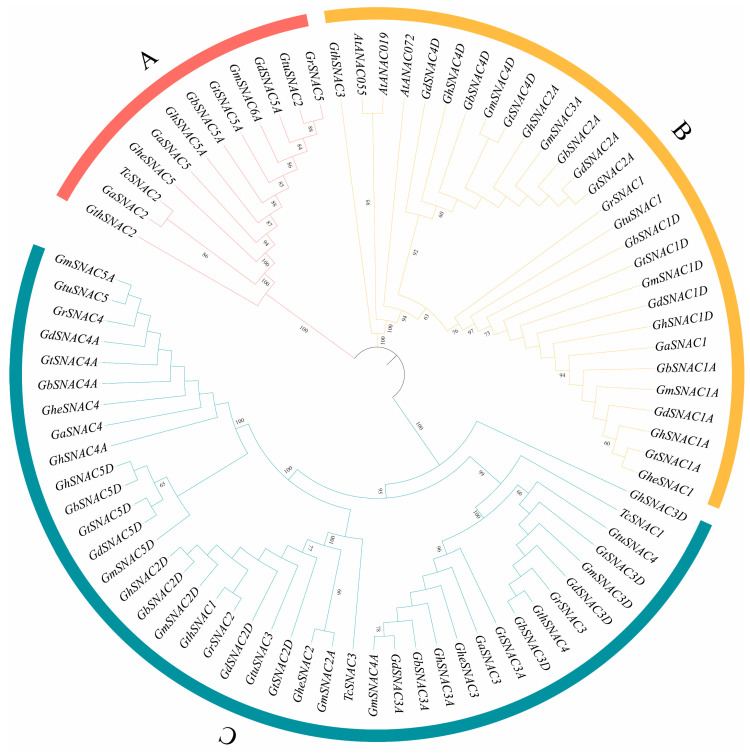
Phylogenetic assessment of SNAC proteins was performed using 81 protein sequences from three species, including *T. cacao*, *A. thaliana*, and 10 *Gossypium* species. These sequences were utilized to construct the neighbor-joining (NJ) tree. Different groups are distinguished by colors: Group A (red), Group B (yellow), and Group C (cyan).

To further characterize the phylogenetic trajectory of SNAC proteins in *Gossypium* and other species, SNAC protein sequences from *A. thaliana*, *T. cacao*, and cotton were subjected to multiple sequence alignment using ClustalW and analyzed using MEGA11 software (v11.0). A phylogenetic tree was constructed via the Neighbor-Joining algorithm (Figure 2). Phylogenetic analysis revealed that SNAC proteins could be classified into three groups. Cotton *SNAC* genes in nearly all clades tended to cluster together, likely due to their relatively conserved functions. Additionally, cotton orthologs from the A genome and At subgenome, as well as from the D genome and Dt subgenome, predominantly formed orthologous pairs at evolutionary branch terminals, indicating closer evolutionary relationships between At-A and Dt-D orthologs in cotton.

**Figure 3:**
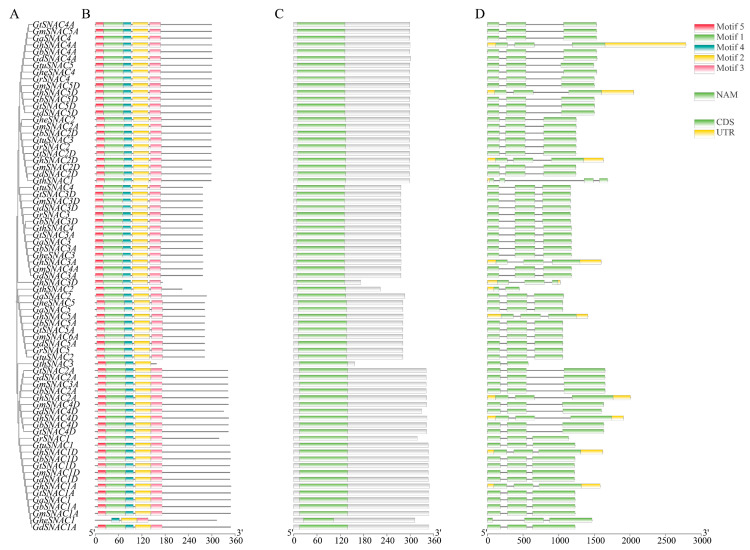
Sequence characteristics of SNAC genes in ten cotton species. (**A**) NJ tree constructed with SNAC proteins from 10 *Gossypium* species; (**B**) Conserved motif analysis of SNAC proteins; (**C**) Characterization of conserved functional domains in SNAC proteins from 10 *Gossypium* species; (**D**) Analysis of gene structures of *SNAC* from 10 *Gossypium* species.

We reconstructed phylogenetic trees from the SNAC protein sequences of 10 species of cotton and analyzed the structural features of the genes (Figure 3A). Consistent with Figure 2, Figure 3A shows that all SNAC proteins in Gossypium were divided into three groups. Figure 3B reveals that nearly all Gossypium SNAC proteins contain motif1-5, with exceptions: GthSNAC3 lacks motif3, and GheSNAC1 lacks motif1 and motif5. This indicates that SNAC proteins are relatively conserved during cotton evolution. Figure 3C demonstrates that all SNAC proteins possess a conserved NAM domain. As shown in Figure 3D, paralogous genes within the same cluster exhibit similar exon/intron distributions, and nearly all SNAC proteins have three CDS regions. This structural similarity provides strong support for exploring the relationship between gene function and evolution. Additionally, several structural variants with distinct exon/intron architectures were identified: GthSNAC1 contains an additional CDS region, while GthSNAC2 and GthSNAC3 have lost one CDS region. These variations may result from evolutionary changes in the SNAC family.

### 2.4. Profiling of Cis-Acting Motifs Within the SNAC Genes Promoter

**Figure 4:**
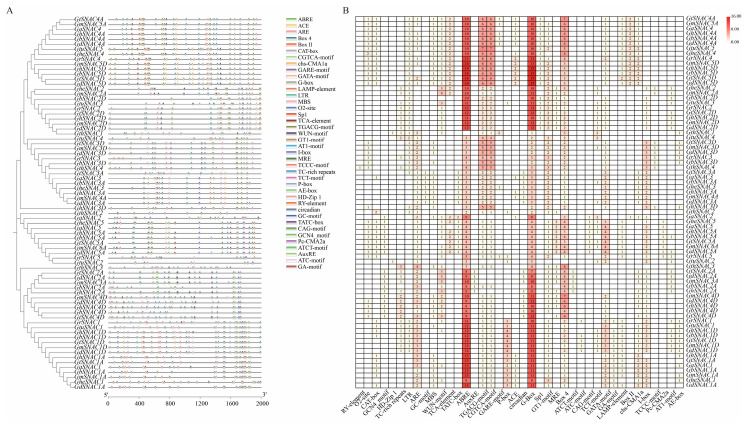
Analysis and prediction of *cis*-regulatory elements in the promoter regions of the SNAC, specifically within the 2000 bp upstream sequences. (**A**) Spatial organization of *cis*-regulatory elements within promoter regions, annotated by color-coded boxes denoting distinct functional motifs. (**B**) Profiling of *cis*-element abundance across gene family members via a heatmap matrix, where gradient shading and numerical labels indicate element-specific counts. These *cis*-regulatory elements were categorized into four functional types: development-related (from *RY-element* to *HD-Zip 1*), environmental stress-related (from *TC-rich repeats* to *WUN-motif*), hormone-responsive (from *TCA-element* to *P-box*), and light-responsive (from *ACE* to *AE-box*).

*Cis*-acting elements located in the promoter region are essential for gene functionality [30]. Most *SNAC* family members exhibit significant differences in both the types and quantity of these elements, indicating a diversification of function within the family [31]. Additionally, members with closer evolutionary relationships share similar element types and quantitative characteristics, as exemplified by *GtSNAC4A*, *GmSNAC5A*, *GaSNAC4*, *GhSNAC4A*, *GbSNAC4A*, *GdSNAC4A*, *GtuSNAC5*, *GheSNAC4*, *GrSNAC4*, *GmSNAC5D*, *GhSNAC5D*, *GbSNAC5D*, *GtSNAC5D* and *GdSNAC5D*. This pattern primarily arises from evolutionary linkage [32]. As shown in Figure 4A,B, a total of 2974 *cis*-regulatory elements were discovered within the cotton *SNAC* gene family, classified as follows: Elements responsive to plant hormones: P-box, GARE-motif, AuxRE, TGACG-motif, TCA-element, TATC-element, CGTCA-motif, and ABRE; Abiotic stress-responsive elements: WUN-motif, LTR, GC-motif, TC-rich repeats, ARE, and MBS; Factors associated with growth and development: HD_Zip1, GCN4_motif, O2-site, RY-element, and CAT-box. Elements that respond to light are the most prevalent, accounting for over 49% of the total. Plant hormone-responsive elements constitute approximately 37%, and abiotic stress-responsive elements about 10%. Despite their relatively small number, abiotic stress-responsive elements are critical for the unique functions of certain genes, particularly in mediating plant resistance to cold stress. These findings indicate that specific *SANC* genes have unique functions in the growth and development of plants by responding to different abiotic stress elements. Overall, the presence of these factors in cotton *SNAC* genes underscores their importance in cold resistance.

### 2.5. Collinearity Analysis of the SNAC Gene Family

**Figure 5:**
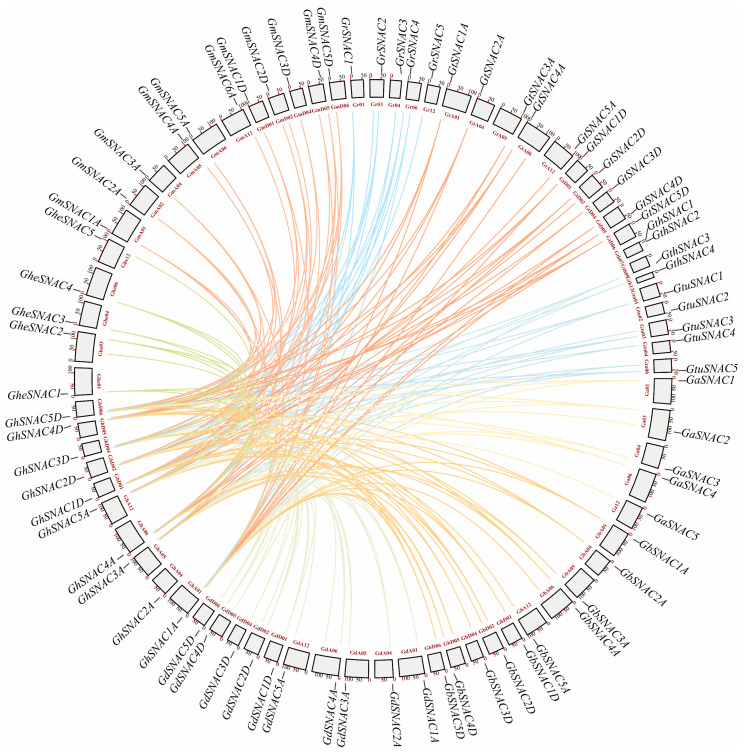
Collinearity between *G. hirsutum* and nine cotton species based on homologous gene pair analysis. In this figure, chromosomes are displayed as gray blocks with labeled names. Collinear genes are linked by different colors: light warm-colored lines indicate collinearity with *G. herbaceum*; light khaki lines with *G. arboreum*; light gray-blue lines with *G. thurberi*; light cyan-blue lines with *G. raimondii*; light blue-gray lines with *G. turneri*; light brownish-yellow lines with *G. barbadense*; light pink-orange lines with *G. tomentosum*; light orange-red lines with *G. mustelinum*; and light taupe lines with *G. darwinii*.

To further explore the evolutionary pattern of *SNAC* genes in upland cotton, we conducted a collinearity analysis comparing *SNAC* genes in upland cotton with those from nine other cotton species. The results revealed 19, 20, 19, 20, 22, 34, 35, 35, and 35 collinear gene pairs in Gth, Ga, Ghe, Gr, Gtu, Gm, Gt, Gb, and Gd, respectively. The widespread collinearity among most genes suggests that *SNAC* genes may be highly conserved in plants (Figure 5). Cross-species collinearity analysis across the 10 cotton species identified a total of 239 collinear pairs among *G. hirsutum* and nine other species. In cotton, the majority *SNAC* gene pairs have undergone segmental duplication occurrences, indicating that segmental duplication serve as the dominant mechanism for the growth of the *SNAC* gene family (Figure 5).

### 2.6. SNAC Genes Expression Pattern Analysis

**Figure 6:**
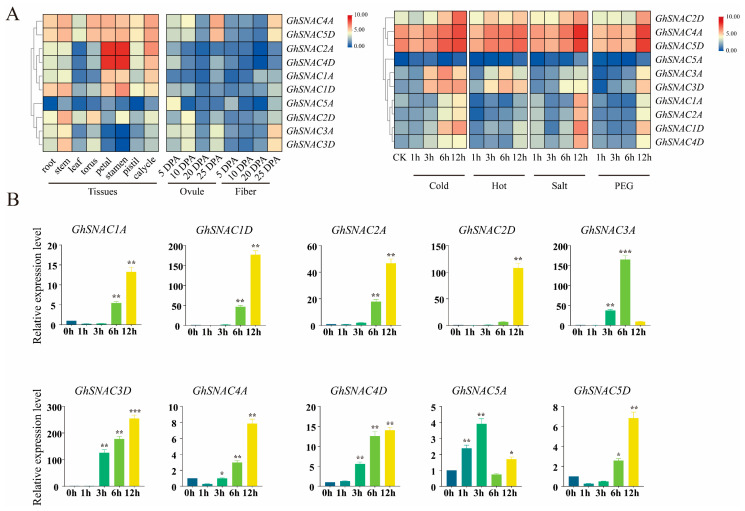
Expression specific to tissue analysis of the *G. hirsutum SNAC*. (**A**) Patterns of Expression *for SNAC* genes across different developmental stages and tissues of cotton, and the level of expression for *GhSNAC* family genes under abiotic stresses at various time intervals. CK indicates the expression level under non-stress conditions. (**B**) Quantitative real-time PCR validation of *GhSNAC* family gene expression levels at various time intervals under cold stress. For the cold stress analysis in Figure 6B, young leaves (the 2nd fully expanded leaf from the top) of 3-leaf-stage cotton seedlings were used. The error bars represent the standard deviation (SD) calculated from three biological replicates. The criteria for significant distinctions are as follows: *, *p* < 0.05, **, *p* < 0.01, and ***, *p* < 0.001.

Based on transcriptome data analysis (PRJNA248163), significant differences were observed in the expression trends of the *SNAC* transcription factor family across distinct development stages and tissues in cotton. Notably, distinct functional divergence exists among different genes within the same family, indicating that *SNAC* genes exhibit obvious tissue-specific expression patterns (Figure 6A). For individual genes, although their expression levels vary significantly across tissues, their expression abundance remains relatively consistent compared to other family members.

Expression analysis further revealed that cotton’s cold stress defense mechanism may rely on key regulatory genes, likely through the coordinated regulation of multiple genes throughout the plant’s growth and development cycle. During cold stress treatments at different times, the levels of expression for *GhSNAC1A*, *GhSNAC1D*, *GhSNAC2A*, *GhSNAC2D*, *GhSNAC3D*, *GhSNAC4A*, *GhSNAC4D*, and *GhSNAC5D* showed a continuous upward trend. Among these, *GhSNAC3D* exhibited the most dramatic expression change: after 3 h of cold stress, the level of expression was roughly 120 times greater than that of the control group, and this increase reached 250-fold after 12 h of stress (Figure 6B). This indicates that *GhSNAC3D* is essential for regulating role cold stress resistance in cotton.

Additionally, most *SNAC* genes were observed to be up-regulated in response to heat, salinity, and drought stress, indicating that the *SNAC* gene family may play a crucial role in cotton’s adaptation to multiple abiotic stressors.

### 2.7. Subcellular Localization of the GhSNAC3D

**Figure 7:**
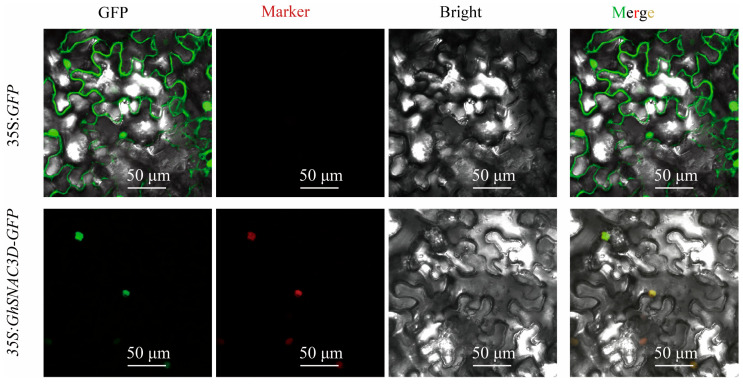
Subcellular distribution of *GhSNAC3D*. The vacant vector (*35S:GFP*), fusion vector (*35S:GhSNAC3D-GFP*), and nuclear marker (*pCAMBIA1300-35S-mCherry-NLS*) were used to infiltrate 4-week-old tobacco plants. Images from left to right correspond to GFP, Marker, Bright, and Merge channels. Green fluorescence indicates GFP signals, red fluorescence represents nuclear-localized marker signals, and yellow fluorescence (Merge) shows the co-localization of GhSNAC3D with the nuclear marker. The scale bar represents 50 µm.

Transcription factors typically exert their functions within the nucleus. In order to investigate the subcellular localization of *GhSNAC3D*, the complete coding of the cloned *GhSNAC3D* gene, with the stop codon removed, was inserted into the *pCAMBIA-1300-GFP* vector, allowing *GhSNAC3D* to be fused with GFP during expression in tobacco leaves. The results confirmed that the empty *pCAMBIA-1300-GFP* vector was expressed throughout the cell, with predominant distribution in the nucleus and cell membrane. In contrast, the GhSNAC3D-GFP fusion protein was exclusively solely to the nucleus, suggesting that *GhSNAC3D* is both expressed and functions within that cellular compartment (Figure 7).

### 2.8. GhSNAC3D Silenced Cotton Plants Showed High Sensitivity to Cold Stress

**Figure 8:**
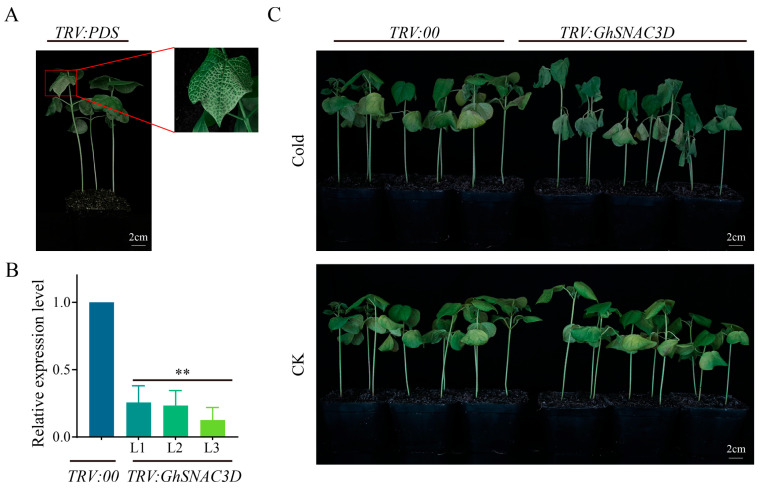
Identification and phenotypic observation of cotton via VIGS. (**A**) Albino phenotype of the *TRV:PDS* treatment group, scale bar: 2 cm; (**B**) *GhSNAC3D* expression levels in silenced plants, significant marks indicate comparisons between L1/L2/L3 and the *TRV:00* control group; (**C**) *TRV:GhSNAC3D* plant phenotype analysis, scale bar: 2 cm. The error bars represent the standard deviation (SD) derived from three biological replicates. The criteria for significant differences are as follows: **, *p* < 0.01.

In order to investigate the function of the *GhSNAC3D* gene in cotton cold stress resistance, we employed VIGS, which is a simple and efficient transient expression method, for functional verification in cotton. As shown in Figure 8C, cotton plants in the *TRV:00* and *TRV:GhSNAC3D* groups showed similar growth, whereas leaves in the *TRV:PDS* group (positive control) exhibited an obvious albino phenotype (Figure 8A), confirming successful VIGS establishment. To validate the silencing efficiency, qRT-PCR analysis was conducted. Results in Figure 8B revealed that the level of expression for *GhSNAC3D* in the *TRV:GhSNAC3D* group was markedly lower than in the *TRV:00* group, with a silencing efficiency of approximately 70% relative to the control, indicating effective gene silencing.

To evaluate the effect of *GhSNAC3D* silencing on resistance to cold stress resistance, plants transformed with *TRV:00* and *TRV:GhSNAC3D* were subjected to cold stress at 4 °C under a cycle consisting of 16 h of light followed by 8 h of darkness for a duration of 12 h. Plants were subjected to cold stress at 4 °C for 12 h starting at the beginning of the 16 h light period [6]. As shown in Figure 8C, leaves of *TRV:GhSNAC3D* plants showed more severe wilting compared to *TRV:00* plants. The finding indicates that *GhSNAC3D* has a positive regulatory function in enhancing cotton cold stress resistance: reduced *GhSNAC3D* expression leads to decreased cold tolerance in cotton.

## 3. Discussion

*SNAC* family is classified under the category of transcription factors that are specific to plants, which is found in *Malus domestica*, *Zea mays*, *Medicago sativa*, *Casuarina equisetifolia*, *Oryza sativa*, *Solanum lycopersicum*, *Arabidopsis thaliana*, *Populus euphratica, Brachypodium distachyon* and other plants [33,34,35,36]. Increasing studies have confirmed that *SNAC* transcription factor family orchestrates developmental processes and serves as a central regulator in biotic/abiotic stress responses by modulating downstream target genes [37]. However, comprehensive investigations into the genome-wide identification, structural characterization, and low-temperature stress response mechanisms of the cotton *SNAC* gene family remain limited. This study implemented an integrated genome-wide identification and expression profiling of the *SNAC* family in cotton, elucidating their structural features and evolutionary relationships while examining the expression dynamics of *GhSNAC* genes under abiotic stress conditions.

Genome-wide analysis successfully characterized the *SNAC* transcription factor family in cotton, with approximately 75 *SNAC* genes annotated in the cotton genome across multiple studies (Table S1). This quantitative distribution resembles the conserved nature of *SNAC* family members across plant species, there are 18 stress-responsive *BdSNACs* in *Brachypodium distachyon* [38], while *Medicago sativa* contains 14 *MsSNACs* in [39], and *Malus domestica* has 17 *MdSNACs* [40]. Phylogenetic analysis, based on a co-constructed tree with *SNAC* genes from *A. thaliana* and *T. cacao*, classified cotton *SNACs* into three evolutionary groups, with Group C containing the largest number of genes (Figure 2), consistent with the taxonomic pattern of *A. thaliana SNACs* [41]. Members of the same subgroup demonstrate remarkable conservation in sequence and structural features, indicating functional conservation within the subgroup (Figure 3); while distinct gene structures and conserved motifs between subgroups suggest that functional differentiation may be due to specific variations in evolutionary processes [42]. Physicochemical property analysis revealed that all cotton SNAC family proteins are hydrophilic (Table S1), consistent with findings in *A. thaliana*, *Casuarina equisetifolia*, and *Malus domestica* [28,41,43]. Collinearity analysis identified 239 collinear gene pairs in cotton (Figure 5), with most originating from segmental duplication events. This indicates that the expansion of the *SNAC* gene family during cotton evolution is predominantly driven by segmental duplication events, which dominated multiple whole-genome duplication episodes. Such a duplication pattern may enhance plant adaptability to environmental stress through functional redundancy while improving genomic plasticity, which aligns with the evolutionary strategy of plants coping with stress [44,45].

Regulation of transcriptional plays a vital role in enabling plants to cope with abiotic stress, as *cis*-acting elements in gene promoters can activate or silence the activity of transcription factors in response to varying abiotic stressors [46]. The type and number of *cis*-acting elements are crucial determinants in the regulation of gene expression and the transcriptional process [46]. This study found that the regulatory regions of cotton *SNAC* genes harbor numerous *cis*-elements responsive to stress as well as hormone-responsive elements. This indicates their potential significance in mediating responses to abiotic stress and in hormonal regulatory pathways within cotton (Figure 4). Notably, similar *cis*-element distribution patterns have been observed in the promoter regions of *SNAC* genes in other plant species, supporting conserved regulatory mechanisms [47]. This convergent evolution may have formed during the long-term adaptation of *Gossypium* species to various environmental cold stress pressures [48]. Transcriptome data from stress response analysis showed that several *GhSNAC* genes exhibit distinct expression changes under cold stress (4 °C) (Figure 6). Further analysis revealed that most stress-responsive *SNAC* genes cluster in the same subgroup in the phylogenetic tree, consistent with findings in *A. thaliana* and *Oryza sativa* [49]. Among them, *GhSNAC3D* was particularly noteworthy: not only was it the most strongly induced by cold stress, but it also exhibited the highest expression level among all *GhSNAC* genes, which strongly suggested that *GhSNAC3D* has a special function in the regulation of the response to cold stress in cotton. To validate its function, *GhSNAC3D*-silenced cotton plants were generated, and cold tolerance assays were performed. Phenotypic analysis showed that, compared with wild-type (WT) plants, *GhSNAC3D*-silenced plants exhibited significantly reduced cold tolerance (Figure 6).

This research conducted a comprehensive analysis of the evolutionary traits and functional diversity of the *SNAC* in cotton, and combined with VIGS technology verification, highlighted the pivotal function of *GhSNAC3D* in the response to cold stress. In the future, transgenic lines can be constructed by transgenic technology to further explore the functional redundancy and synergistic mechanism of members of different subgroups. Simultaneously, it offers a theoretical foundation for the breeding of crops with resistance to stress.

## 4. Materials and Methods

### 4.1. Plant Materials and Experimental Treatments

The plant material utilized in this experiment is *G. hirsutum cv. TM-1* and the seeds were purchased from the Institute of Cotton Research, Chinese Academy of Agricultural Sciences. Cotton seeds were sulfuric acid delinting and sown in nutrient bowl filled with nutrient soil: vermiculite: perlite = 3:1:1. Plant was maintained in controlled environment chambers with thermostatic regulation at 25 °C, 16 h light/8 h dark, and 70% relative humidity. Nutrient management was carried out using Hoagland nutrient solution (watered once a week until 3 days before stress treatment, and purchased from Tiangen Biotech BeiJing Co., Ltd. (Beijing, China)). Fifteen-day-old vegetative-stage plantlets were exposed to 4 °C treatment in growth chambers maintaining standardized photoperiodic parameters. Samples of leaves were collected at various intervals (0 h, 1 h, 3 h, 6 h and 12 h following stress induction) after treatment. Then freeze-fix immediately and maintain RNA integrity at −80 °C until nucleic acid extraction.

### 4.2. Characterization of Members of the SNAC Family

Genome datasets for ten *Gossypium* species (*G. herbaceum*, WHU, A1; *G. arboretum*, CRI, A2; *G. thurberi*, ISU, D1; *G. raimondii*, HAU, D5; *G. turneri*, NSF, D10; *G. barbadense*, ZJU, AD2; *G. mustelinum*, JGI, AD4; *G. tomentosum*, HGS, AD3; *G. hirsutum*, JGI, AD1; *G. darwinii*, HGS, AD5) were obtained from the Cotton Multiomics Database [50]. The homologous SNAC protein sequences from *Arabidopsis* were retrieved from the TAIR database [51]. The SNAC protein sequences from *Arabidopsis* as query sequences (the sequences listed in Supplementary Table S4), the BLAST program package within TBtools (v2.210) was utilized to conduct BLASTP alignment searches on the completed local whole—genome databases of 10 cotton species [52]. The E-value threshold was set to 1.0 × 10^−20^ to reduce false positives and obtain the dataset of SNAC proteins. Only candidate genes with ≥60% homology to SNAC subfamily genes [53]. Predict and analyze the conserved domains of the preliminarily identified candidate homologous gene pairs in the InterProScan database and SMART database [54,55]. Eliminate gene sequences that either lack or do not include the conserved domains typical of the *SNAC* family, and further screen to obtain the final family members. In addition, using the pI/Mw tool from ExPASY was utilized to determine the physicochemical properties of each gene product, such as molecular weight (MW) and isoelectric point (pI).

### 4.3. Phylogenetic Investigation

The MEGA 11 (Molecular Evolutionary Genetics Analysis software, Version 11.0) software is used for phylogenetic research. The ClustalW method is employed to align the protein sequences of SNAC among *T. cacao*, *A. thaliana*, and ten *Gossypium* species. Then, these protein sequences are utilized to create a phylogenetic tree through the Neighbor-Joining (NJ) algorithm, with parameters set to 1000 bootstrap replicates and genetic distance calculated using the Poisson correction model. The generated tree is visualized using the iTOL website [56].

### 4.4. Chromosomal Distribution of SNAC Gene

Spatial distributions of these genes across ten Gossypium species were visualized on chromosomes using the MG2C (online tool for Multiple Sequence Alignment and Phylogenetic Tree Construction, Version 2.0) platform [57].

### 4.5. Genomic Architecture, Conserved Motifs, and Protein Domains Analyses

Conserved motifs within protein sequences were predicted through the use of the online MEME database [58], and conserved domains were predicted via the online CD-Search database. The genomic architecture, conserved motifs, and protein domains were visualized via TBtools.

### 4.6. Investigation of Cis-Acting Elements in the Promoter Region Upstream of the Land Cotton SNAC Gene

*Cis*-regulatory elements located in the 2000 bp upstream promoter regions of *Gossypium SNAC* family were predicted and examined using the PlantCARE database with visualization performed via TBtools [59].

### 4.7. Collinearity Analysis

Collinearity analysis of ten *Gossypium SNAC* genes was performed using the One Step MCScanX module in TBtools.

### 4.8. GhSNAC Expression Pattern Analysis

Transcriptomic datasets encompassing multiple tissues and abiotic stress conditions of *G hirsutum* were analyzed to profile *SNAC* gene expression patterns [60]. The transcriptomic dataset analyzed in this study was obtained from a public database with a login number of PRJNA248163 obtained from the NCBI SRA database. Heatmaps were generated using the Heatmap module in TBtools, with transcriptomic data standardized by Log_2_(FPKM+1).

### 4.9. Quantitative Reverse Transcription Polymerase Chain Reaction (RT-qPCR) Analysis

Total RNA was isolated from cotton leaves and purified using the RNAprep Pure Polysaccharide Polyphenol Plant Total RNA Extraction Kit (TIANGEN lot: A0516A). *GhSNACs* expression in these tissues was analyzed via quantitative real-time PCR (RT-qPCR), with cotton *GhUBQ6* serving as internal controls [61]. Briefly, cDNA was reverse-transcribed from total RNA and utilized as template for RT-qPCR with gene-specific primers. Reactions were conducted using SYBR-Green real-time PCR premix following the manufacturer’s protocol, and relative expression levels were calculated via the 2^−ΔΔCt^ method [62].

### 4.10. Subcellular Localization

*GhSNAC3D* was amplified via PCR and cloned into the *pCAMBIA1300-GFP (35S:GFP)* expression vector through homologous recombination, yielding a *pCAMBIA1300-GFP-GhSNAC3D (35S:GhSNAC3D-GFP)* fusion construct (primers listed in Supplementary Table S2). The constructed vector was introduced into *Agrobacterium tumefaciens* GV3101 and subsequently infiltrated into the leaves of 4-week-old *Nicotiana benthamiana*, followed by dark incubation for 48 h. Fluorescence emissions from the leaf epidermis of *N. benthamiana* were visualized via a confocal microscope (Nikon Corporation, Tokyo, Japan). The study employed *pCAMBIA1300-35S-mCherry-NLS* (Puint, Xianning, China) as a nuclear marker for the cells.

### 4.11. Construction and Transformation of Cotton Vectors

To construct cotton silencing vectors, specific fragments (300 bp in length) of the *GhSNAC3D* and *GhPDS* genes were selected to avoid high homology with homologous genes (primers listed in Supplementary Table S2). These fragments were cloned into the *pTRV2* vector. The constructed silencing vectors were then introduced into *Agrobacterium* LBA4404 via electroporation, and positive strains were verified by PCR [63]. The transformed *Agrobacterium* was cultured to the logarithmic growth phase, and the infection concentration was adjusted to an appropriate level (OD_600_ = 1.5). The *pTRV2* and *pTRV1* empty vectors, positive control, and *Ptrv:GhSNAC3D* were mixed at a 1:1 ratio and infiltrated into cotton leaves by injection. Infected cotton plants were incubated in the dark overnight and subsequently transferred to a cotton cultivation room at a temperature of 25 °C, with a light cycle of 16 h on and 8 h off for cultivation.

### 4.12. Data Statistics

Statistical evaluations were conducted utilizing GraphPad Prism (v10.1.2). Duncan’s multiple-range test was employed to assess differences between measurements across time points or treatment groups. Significance thresholds were defined as follows: *p* > 0.05 (not significant), *p* < 0.01 (highly significant), and *p* < 0.05 (statistically significant).

## 5. Conclusions

This study systematically identified members of the cotton *SNAC* family and clarified the core role of *GhSNAC3D* in cold stress response, thus providing candidate genes for cotton stress-resistant molecular breeding. At present, only the phenotypic effects of the gene have been verified, and *GhSNAC3D’s* downstream target genes and specific molecular mechanisms still need to be further analyzed through experiments such as yeast one-hybrid and ChIP-seq. In the future, we will obtain stably overexpressed plants through genetic transformation, analyze their agronomic traits under field stress conditions, and explore the interaction network between *GhSNAC3D* and other stress-resistant genes.

## Data Availability

Data are contained within the article and Supplementary Materials.

## Supplementary Materials

The following supporting information can be downloaded at https://www.mdpi.com/article/10.3390/plants14182894/s1, Table S1: Biochemical Characterization of *SNAC* gene family members; Table S2: Primers used in this experiment; Table S3: The Connection between the Database and the Online Website in This Study; Table S4: The sequence information for the Arabidopsis SNAC proteins.
